# 1,1,2,2-Tetra­kis(1,3-benzothia­zol-2-yl)ethene chloro­form disolvate

**DOI:** 10.1107/S1600536810032526

**Published:** 2010-08-21

**Authors:** Tesfamariam K. Hagos, Stefan D. Nogai, Liliana Dobrzańska, Stephanie Cronje, Helgard G. Raubenheimer

**Affiliations:** a Department of Chemistry, University of Stellenbosch, Private Bag X1, Matieland, South Africa

## Abstract

The asymmetric unit of the title solvate, C_30_H_16_N_4_S_4_·2CHCl_3_, contains one half-molecule of tetrakis(2-benzothiazolyl)ethene, the complete molecule being generated by inversion symmetry, and one molecule of chloroform. Pairs of the benzothia­zole rings attached to the same carbon atom are almost perpendicular to each other, with an angle between planes of 85.74 (4)°. In the crystal, weak C—H⋯N and C—H⋯Cl interactions generate a three-dimensional network.

## Related literature

For our recent studies on gold chemistry with heterocycles, see: Strasser *et al.* (2010 ▶); Gabrielli *et al.* (2009 ▶). For the crystal structure of the reduced form of the title compound, see: Boga *et al.* (1999 ▶). For bond lengths of benzothia­zole rings in related compounds, see: Pavlović *et al.* (2007 ▶); Pindinelli *et al.* (2007 ▶); Cox *et al.* (1993 ▶). For details on the cut-off applied for the C—H⋯Cl inter­actions, see: Brammer *et al.* (2001 ▶). For the synthesis of AuCl(PPh_3_), see: Bruce *et al.* (1989 ▶).
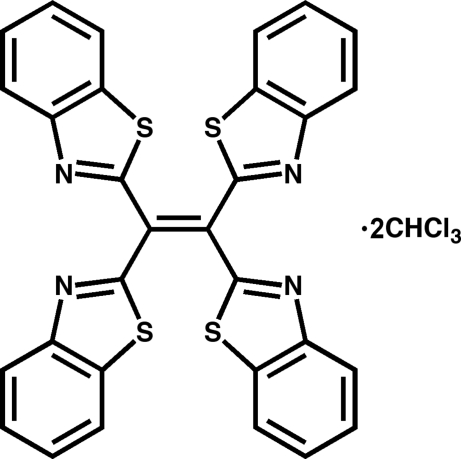


## Experimental

### 

#### Crystal data

C_30_H_16_N_4_S_4_·2CHCl_3_
*M* *_r_* = 799.44
Monoclinic, 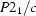

*a* = 9.955 (2) Å
*b* = 16.299 (3) Å
*c* = 11.569 (2) Å
β = 115.61 (3)°
*V* = 1692.8 (6) Å^3^
*Z* = 2
Mo *K*α radiation
μ = 0.79 mm^−1^
*T* = 100 K
0.35 × 0.25 × 0.15 mm

#### Data collection

Bruker APEX CCD area-detector diffractometer
Absorption correction: multi-scan (*SADABS*; Sheldrick, 1997 ▶) *T*_min_ = 0.770, *T*_max_ = 0.891
19051 measured reflections
4018 independent reflections
3704 reflections with *I* > 2σ(*I*)
*R*_int_ = 0.037

#### Refinement

*R*[*F* ^2^ > 2σ(*F* ^2^)] = 0.038
*wR*(*F* ^2^) = 0.092
*S* = 1.07
4018 reflections
208 parameters
H-atom parameters constrained
Δρ_max_ = 0.83 e Å^−3^
Δρ_min_ = −0.62 e Å^−3^

### 

Data collection: *SMART* (Bruker, 2001 ▶); cell refinement: *SAINT* (Bruker, 2002 ▶); data reduction: *SAINT*; program(s) used to solve structure: *SHELXS97* (Sheldrick, 2008 ▶); program(s) used to refine structure: *SHELXL97* (Sheldrick, 2008 ▶); molecular graphics: *Mercury* (Macrae *et al.*, 2008 ▶); software used to prepare material for publication: *SHELXL97*.

## Supplementary Material

Crystal structure: contains datablocks I, global. DOI: 10.1107/S1600536810032526/hg2698sup1.cif

Structure factors: contains datablocks I. DOI: 10.1107/S1600536810032526/hg2698Isup2.hkl

Additional supplementary materials:  crystallographic information; 3D view; checkCIF report

## Figures and Tables

**Table 1:** Hydrogen-bond geometry (Å, °)

*D*—H⋯*A*	*D*—H	H⋯*A*	*D*⋯*A*	*D*—H⋯*A*
C20—H20⋯N12^i^	1.00	2.23	3.148 (3)	153
C6—H6⋯Cl23^ii^	0.95	2.90	3.690 (2)	141
C7—H7⋯N3^iii^	0.95	2.66	3.300 (3)	125
C8—H8⋯N3^iii^	0.95	2.70	3.306 (2)	122
C17—H17⋯N3^iv^	0.95	2.71	3.565 (3)	149

Symmetry codes: (i) 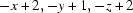; (ii) 
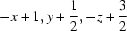
; (iii) 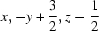; (iv) 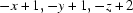.

**Fig. 1.**
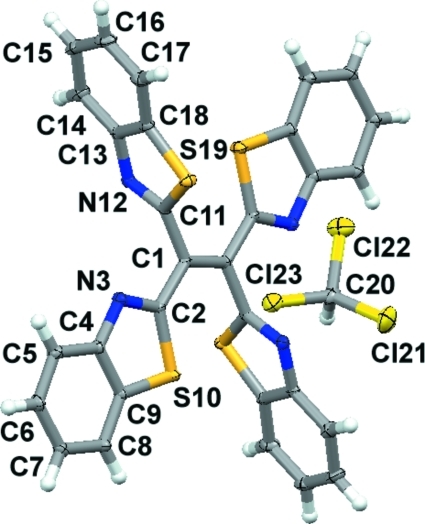
The molecular structure of the title molecule, with the atom-numbering scheme. Displacement ellipsoids are drawn at the 50% probability level. Unlabelled atoms are related to the labelled ones by the symmetry operation (i) 2 - x, 1 - y, 2 - z.

**Fig. 2.**
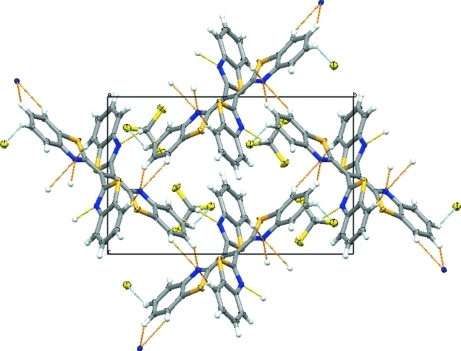
Representation of the packing viewed down the a axis; weak C—H···N3 interactions are indicated by orange dashed lines, C20—H20···N12 and C6—H6···Cl23 by yellow and light blue dashed lines, respectively.

## supplementary crystallographic information

### Comment

The title chloroform solvate was isolated during ongoing research involving gold complexes and N-heterocycles (Strasser *et al.* 2010; Gabrielli *et al.* 2009) as a product of oxidation and dimerization of bis(2-benzothiazolyl)methane with chloroauric acid. The asymmetric unit consists of half of the tetrakis(2-benzothiazolyl)ethene molecule (the inversion centre generates the other half) and one molecule of chloroform (Fig. 1). The crystal structure of the reduced form of this compound, namely tetrakis(2-benzothiazolyl)ethane dichloromethane solvate was reported earlier by Boga *et al.* (1999). The conformations adopted by the benzothiazole rings differ significantly in the two molecules. In the reduced compound the pairs of the benzothiazole rings attached to the same carbon were more or less co-planar whereas in the compound presented here the planes of the corresponding rings are almost perpendicular to each other with an angle between the planes of 85.74 (4)°. The bond length of 1.359 (3) Å for C1—C1^i^ (symmetry operation (i): 2 - *x*, -*y* + 1, 2 - *z*) confirms the formation of a double bond. As could be expected, in the reduced form the corresponding (single) bond is *ca* 0.1 Å longer. The bond lengths for the benzothiazole rings are in good agreement with previously reported values (Pavlović *et al.*, 2007; Pindinelli *et al.*, 2007; Cox *et al.*, 1993). N3 from one of the benzothiazole rings acts as an acceptor of three C—H···N weak hydrogen bonds orginating from neighbouring benzothiazole rings (Table 1) resulting in the formation of a three-dimensional assembly. The nitrogen atoms from another benzothiazole ring, namely N12, participate in weak interactions between chloroform and tetrakis(2-benzothiazolyl)ethene molecules (C20—H20···N12^i^ with a C···N distance of 3.148 (3) Å). Those together with interactions between C6—H6···Cl23^ii^ atoms (symmetry operation (ii): -*x* + 1, *y* + 1/2, 3/2 - *z*) with a C···Cl distance of 3.690 (2) (Brammer *et al.*, 2001) further support the resulting packing (Fig. 2).

### Experimental

A solution of bis(2-benzothiazolyl)methane in THF was treated with an equimolar quantity of n-BuLi in n-hexane at 203 K. The resulting blue fluorescent mixture was treated with a solution of one molar equivalent of AuCl(PPh_3_) (Bruce *et al.*, 1989) in THF and stirred for 2 h. The solvent was removed under vacuum. Crystallization of the residue from a chloroform solution layered with n-hexane at 253 K afforded a mixture of yellow and orange crystals. Single crystal X-ray studies of the yellow crystals showed the oxidative dimerization of the bis(2-benzothiazolyl)methane to yield the title compound.

### Refinement

All H atoms were positioned geometrically, with C—H = 0.95 and 1 Å for aromatic and chloroform H, respectively, and constrained to ride on their parent atoms with *U*_iso_(H) = 1.2*U*_eq_(C).

### Crystal data

**Table:**

C_30_H_16_N_4_S_4_·2CHCl_3_	*F*(000) = 808
*M**_r_* = 799.44	*D*_x_ = 1.568 Mg m^−^^3^
Monoclinic, *P*2_1_/*c*	Mo *K*α radiation, λ = 0.71073 Å
Hall symbol: -P 2ybc	Cell parameters from 2580 reflections
*a* = 9.955 (2) Å	θ = 2.7–27.5°
*b* = 16.299 (3) Å	µ = 0.79 mm^−^^1^
*c* = 11.569 (2) Å	*T* = 100 K
β = 115.61 (3)°	Block, yellow
*V* = 1692.8 (6) Å^3^	0.35 × 0.25 × 0.15 mm
*Z* = 2	

### Data collection

**Table:**

Bruker APEX CCD area-detector diffractometer	4018 independent reflections
Radiation source: fine-focus sealed tube	3704 reflections with *I* > 2σ(*I*)
graphite	*R*_int_ = 0.037
ω scans	θ_max_ = 28.2°, θ_min_ = 2.3°
Absorption correction: multi-scan (*SADABS*; Sheldrick, 1997)	*h* = −12→12
*T*_min_ = 0.770, *T*_max_ = 0.891	*k* = −21→21
19051 measured reflections	*l* = −15→14

### Refinement

**Table:**

Refinement on *F*^2^	Primary atom site location: structure-invariant direct methods
Least-squares matrix: full	Secondary atom site location: difference Fourier map
*R*[*F*^2^ > 2σ(*F*^2^)] = 0.038	Hydrogen site location: inferred from neighbouring sites
*wR*(*F*^2^) = 0.092	H-atom parameters constrained
*S* = 1.07	*w* = 1/[σ^2^(*F*_o_^2^) + (0.0425*P*)^2^ + 1.4464*P*] where *P* = (*F*_o_^2^ + 2*F*_c_^2^)/3
4018 reflections	(Δ/σ)_max_ = 0.001
208 parameters	Δρ_max_ = 0.83 e Å^−^^3^
0 restraints	Δρ_min_ = −0.62 e Å^−^^3^

### Special details

**Table:**

Geometry. All e.s.d.'s (except the e.s.d. in the dihedral angle between two l.s. planes) are estimated using the full covariance matrix. The cell e.s.d.'s are taken into account individually in the estimation of e.s.d.'s in distances, angles and torsion angles; correlations between e.s.d.'s in cell parameters are only used when they are defined by crystal symmetry. An approximate (isotropic) treatment of cell e.s.d.'s is used for estimating e.s.d.'s involving l.s. planes.
Refinement. Refinement of *F*^2^ against ALL reflections. The weighted *R*-factor *wR* and goodness of fit *S* are based on *F*^2^, conventional *R*-factors *R* are based on *F*, with *F* set to zero for negative *F*^2^. The threshold expression of *F*^2^ > σ(*F*^2^) is used only for calculating *R*-factors(gt) *etc*. and is not relevant to the choice of reflections for refinement. *R*-factors based on *F*^2^ are statistically about twice as large as those based on *F*, and *R*- factors based on ALL data will be even larger.

### Fractional atomic coordinates and isotropic or equivalent isotropic displacement parameters (Å^2^)

**Table:**

	*x*	*y*	*z*	*U*_iso_*/*U*_eq_	
C1	0.94075 (18)	0.52372 (10)	0.99285 (15)	0.0127 (3)	
C2	0.87364 (18)	0.58940 (10)	0.89861 (15)	0.0123 (3)	
N3	0.76258 (16)	0.63137 (9)	0.90007 (14)	0.0143 (3)	
C4	0.71780 (18)	0.69236 (10)	0.80832 (16)	0.0139 (3)	
C5	0.6029 (2)	0.74885 (11)	0.78653 (17)	0.0183 (3)	
H5	0.5477	0.7473	0.8360	0.022*	
C6	0.57244 (19)	0.80648 (11)	0.69183 (17)	0.0179 (3)	
H6	0.4950	0.8451	0.6761	0.021*	
C7	0.65214 (19)	0.80981 (10)	0.61813 (16)	0.0167 (3)	
H7	0.6274	0.8502	0.5529	0.020*	
C8	0.76687 (19)	0.75521 (11)	0.63852 (17)	0.0171 (3)	
H8	0.8220	0.7577	0.5890	0.020*	
C9	0.79844 (18)	0.69662 (10)	0.73415 (16)	0.0139 (3)	
S10	0.93325 (5)	0.62064 (3)	0.78321 (4)	0.01696 (11)	
C11	0.86666 (17)	0.51295 (10)	1.08024 (16)	0.0122 (3)	
N12	0.91748 (16)	0.54510 (9)	1.19354 (14)	0.0138 (3)	
C13	0.82944 (19)	0.52252 (10)	1.25436 (16)	0.0146 (3)	
C14	0.8538 (2)	0.54653 (12)	1.37835 (17)	0.0203 (4)	
H14	0.9341	0.5818	1.4278	0.024*	
C15	0.7579 (2)	0.51753 (13)	1.42658 (18)	0.0248 (4)	
H15	0.7733	0.5328	1.5106	0.030*	
C16	0.6383 (2)	0.46595 (13)	1.35398 (19)	0.0247 (4)	
H16	0.5749	0.4465	1.3902	0.030*	
C17	0.6109 (2)	0.44282 (12)	1.23095 (18)	0.0210 (4)	
H17	0.5289	0.4085	1.1814	0.025*	
C18	0.70809 (19)	0.47171 (10)	1.18186 (16)	0.0153 (3)	
S19	0.70563 (5)	0.45367 (3)	1.03337 (4)	0.01547 (11)	
C20	0.7989 (2)	0.34163 (12)	0.71325 (18)	0.0233 (4)	
H20	0.8939	0.3669	0.7209	0.028*	
Cl21	0.72698 (8)	0.27883 (4)	0.57692 (6)	0.04317 (16)	
Cl22	0.83624 (6)	0.28346 (4)	0.85199 (5)	0.03533 (14)	
Cl23	0.67054 (6)	0.42068 (3)	0.69781 (5)	0.02985 (13)	

### Atomic displacement parameters (Å^2^)

**Table:**

	*U*^11^	*U*^22^	*U*^33^	*U*^12^	*U*^13^	*U*^23^
C1	0.0136 (7)	0.0128 (7)	0.0119 (7)	−0.0026 (6)	0.0058 (6)	−0.0015 (6)
C2	0.0138 (7)	0.0128 (7)	0.0117 (7)	−0.0004 (6)	0.0068 (6)	0.0001 (6)
N3	0.0143 (7)	0.0146 (7)	0.0149 (7)	0.0002 (5)	0.0070 (6)	0.0014 (5)
C4	0.0138 (8)	0.0140 (8)	0.0141 (7)	−0.0007 (6)	0.0062 (6)	−0.0002 (6)
C5	0.0184 (8)	0.0198 (8)	0.0200 (8)	0.0040 (7)	0.0115 (7)	0.0027 (7)
C6	0.0171 (8)	0.0161 (8)	0.0201 (8)	0.0044 (6)	0.0077 (7)	0.0027 (7)
C7	0.0176 (8)	0.0146 (8)	0.0155 (8)	0.0004 (6)	0.0050 (7)	0.0029 (6)
C8	0.0177 (8)	0.0183 (8)	0.0172 (8)	0.0007 (7)	0.0094 (7)	0.0032 (7)
C9	0.0138 (8)	0.0130 (7)	0.0155 (8)	0.0016 (6)	0.0068 (6)	0.0013 (6)
S10	0.0184 (2)	0.0190 (2)	0.0184 (2)	0.00666 (16)	0.01248 (17)	0.00728 (16)
C11	0.0116 (7)	0.0108 (7)	0.0140 (7)	0.0012 (6)	0.0054 (6)	0.0022 (6)
N12	0.0138 (7)	0.0142 (7)	0.0143 (7)	0.0017 (5)	0.0070 (6)	0.0013 (5)
C13	0.0144 (8)	0.0144 (8)	0.0157 (8)	0.0037 (6)	0.0074 (6)	0.0027 (6)
C14	0.0202 (9)	0.0250 (9)	0.0164 (8)	0.0022 (7)	0.0085 (7)	0.0005 (7)
C15	0.0273 (10)	0.0347 (11)	0.0168 (9)	0.0062 (8)	0.0136 (8)	0.0035 (8)
C16	0.0230 (9)	0.0324 (10)	0.0256 (10)	0.0055 (8)	0.0169 (8)	0.0095 (8)
C17	0.0170 (8)	0.0250 (9)	0.0234 (9)	0.0002 (7)	0.0109 (7)	0.0054 (7)
C18	0.0152 (8)	0.0160 (8)	0.0158 (8)	0.0026 (6)	0.0077 (7)	0.0031 (6)
S19	0.0146 (2)	0.0181 (2)	0.0143 (2)	−0.00416 (15)	0.00682 (16)	−0.00119 (15)
C20	0.0240 (9)	0.0256 (10)	0.0243 (9)	−0.0075 (7)	0.0143 (8)	−0.0064 (7)
Cl21	0.0606 (4)	0.0355 (3)	0.0312 (3)	−0.0043 (3)	0.0178 (3)	−0.0158 (2)
Cl22	0.0303 (3)	0.0464 (3)	0.0305 (3)	0.0025 (2)	0.0143 (2)	0.0093 (2)
Cl23	0.0364 (3)	0.0275 (3)	0.0333 (3)	0.0002 (2)	0.0222 (2)	−0.00071 (19)

### Geometric parameters (Å, °)

**Table:**

C1—C1^i^	1.359 (3)	C11—S19	1.7453 (17)
C1—C2	1.467 (2)	N12—C13	1.390 (2)
C1—C11	1.497 (2)	C13—C14	1.403 (2)
C2—N3	1.306 (2)	C13—C18	1.405 (2)
C2—S10	1.7546 (16)	C14—C15	1.380 (3)
N3—C4	1.380 (2)	C14—H14	0.9500
C4—C5	1.404 (2)	C15—C16	1.402 (3)
C4—C9	1.408 (2)	C15—H15	0.9500
C5—C6	1.374 (2)	C16—C17	1.381 (3)
C5—H5	0.9500	C16—H16	0.9500
C6—C7	1.394 (2)	C17—C18	1.398 (2)
C6—H6	0.9500	C17—H17	0.9500
C7—C8	1.385 (2)	C18—S19	1.7327 (18)
C7—H7	0.9500	C20—Cl21	1.753 (2)
C8—C9	1.391 (2)	C20—Cl22	1.760 (2)
C8—H8	0.9500	C20—Cl23	1.769 (2)
C9—S10	1.7315 (17)	C20—H20	1.0000
C11—N12	1.294 (2)		
			
C1^i^—C1—C2	126.72 (19)	C1—C11—S19	120.63 (12)
C1^i^—C1—C11	120.41 (19)	C11—N12—C13	110.45 (14)
C2—C1—C11	112.86 (14)	N12—C13—C14	125.14 (16)
N3—C2—C1	119.25 (14)	N12—C13—C18	114.94 (15)
N3—C2—S10	115.11 (12)	C14—C13—C18	119.93 (16)
C1—C2—S10	125.61 (12)	C15—C14—C13	118.27 (18)
C2—N3—C4	111.16 (14)	C15—C14—H14	120.9
N3—C4—C5	125.30 (15)	C13—C14—H14	120.9
N3—C4—C9	115.13 (15)	C14—C15—C16	121.31 (18)
C5—C4—C9	119.56 (15)	C14—C15—H15	119.3
C6—C5—C4	118.26 (16)	C16—C15—H15	119.3
C6—C5—H5	120.9	C17—C16—C15	121.31 (17)
C4—C5—H5	120.9	C17—C16—H16	119.3
C5—C6—C7	121.77 (16)	C15—C16—H16	119.3
C5—C6—H6	119.1	C16—C17—C18	117.61 (18)
C7—C6—H6	119.1	C16—C17—H17	121.2
C8—C7—C6	121.06 (16)	C18—C17—H17	121.2
C8—C7—H7	119.5	C17—C18—C13	121.56 (17)
C6—C7—H7	119.5	C17—C18—S19	129.04 (15)
C7—C8—C9	117.57 (16)	C13—C18—S19	109.39 (13)
C7—C8—H8	121.2	C18—S19—C11	88.89 (8)
C9—C8—H8	121.2	Cl21—C20—Cl22	110.44 (11)
C8—C9—C4	121.77 (15)	Cl21—C20—Cl23	109.77 (11)
C8—C9—S10	128.94 (13)	Cl22—C20—Cl23	110.05 (10)
C4—C9—S10	109.29 (12)	Cl21—C20—H20	108.8
C9—S10—C2	89.31 (8)	Cl22—C20—H20	108.8
N12—C11—C1	123.05 (15)	Cl23—C20—H20	108.8
N12—C11—S19	116.31 (13)		
			
C1^i^—C1—C2—N3	−177.7 (2)	C1^i^—C1—C11—N12	81.4 (3)
C11—C1—C2—N3	1.0 (2)	C2—C1—C11—N12	−97.33 (19)
C1^i^—C1—C2—S10	0.1 (3)	C1^i^—C1—C11—S19	−97.5 (2)
C11—C1—C2—S10	178.72 (12)	C2—C1—C11—S19	83.71 (16)
C1—C2—N3—C4	177.65 (14)	C1—C11—N12—C13	−177.85 (14)
S10—C2—N3—C4	−0.34 (18)	S19—C11—N12—C13	1.15 (18)
C2—N3—C4—C5	−178.99 (17)	C11—N12—C13—C14	179.76 (16)
C2—N3—C4—C9	0.1 (2)	C11—N12—C13—C18	−0.3 (2)
N3—C4—C5—C6	179.50 (16)	N12—C13—C14—C15	−178.67 (17)
C9—C4—C5—C6	0.5 (3)	C18—C13—C14—C15	1.4 (3)
C4—C5—C6—C7	0.0 (3)	C13—C14—C15—C16	−0.5 (3)
C5—C6—C7—C8	−0.6 (3)	C14—C15—C16—C17	−0.7 (3)
C6—C7—C8—C9	0.6 (3)	C15—C16—C17—C18	0.9 (3)
C7—C8—C9—C4	−0.1 (3)	C16—C17—C18—C13	−0.1 (3)
C7—C8—C9—S10	−179.85 (14)	C16—C17—C18—S19	179.48 (15)
N3—C4—C9—C8	−179.57 (16)	N12—C13—C18—C17	178.94 (16)
C5—C4—C9—C8	−0.5 (3)	C14—C13—C18—C17	−1.1 (3)
N3—C4—C9—S10	0.23 (19)	N12—C13—C18—S19	−0.69 (18)
C5—C4—C9—S10	179.34 (13)	C14—C13—C18—S19	179.28 (13)
C8—C9—S10—C2	179.45 (17)	C17—C18—S19—C11	−178.54 (17)
C4—C9—S10—C2	−0.33 (13)	C13—C18—S19—C11	1.05 (13)
N3—C2—S10—C9	0.40 (14)	N12—C11—S19—C18	−1.33 (14)
C1—C2—S10—C9	−177.44 (15)	C1—C11—S19—C18	177.70 (14)

Symmetry codes: (i) −*x*+2, −*y*+1, −*z*+2.

### Hydrogen-bond geometry (Å, °)

**Table:**

*D*—H···*A*	*D*—H	H···*A*	*D*···*A*	*D*—H···*A*
C20—H20···N12^i^	1.00	2.23	3.148 (3)	153
C6—H6···Cl23^ii^	0.95	2.90	3.690 (2)	141
C7—H7···N3^iii^	0.95	2.66	3.300 (3)	125
C8—H8···N3^iii^	0.95	2.70	3.306 (2)	122
C17—H17···N3^iv^	0.95	2.71	3.565 (3)	149

Symmetry codes: (i) −*x*+2, −*y*+1, −*z*+2; (ii) −*x*+1, *y*+1/2, −*z*+3/2; (iii) *x*, −*y*+3/2, *z*−1/2; (iv) −*x*+1, −*y*+1, −*z*+2.
